# The cysteinyl leukotriene 2 receptor contributes to all-*trans* retinoic acid-induced differentiation of colon cancer cells

**DOI:** 10.1186/1471-2407-13-336

**Published:** 2013-07-08

**Authors:** Astrid M Bengtsson, Gunilla Jönsson, Cecilia Magnusson, Tavga Salim, Cecilia Axelsson, Anita Sjölander

**Affiliations:** 1 Department of Laboratory Medicine, Cell and Experimental Pathology, Lund University, Malmö University Hospital, SE-205 02, Malmö, Sweden; 2 Present name and address, Astrid Friborg at Berries by Astrid, Stockholm, Sweden

**Keywords:** All-*trans* retinoic acid (ATRA), CysLT_2_R, Leukotriene, Leukotriene receptor, Colon cancer, Inflammation

## Abstract

**Background:**

Cysteinyl leukotrienes (CysLTs) are potent pro-inflammatory mediators that are increased in samples from patients with inflammatory bowel diseases (IBDs). Individuals with IBDs have enhanced susceptibility to colon carcinogenesis. In colorectal cancer, the balance between the pro-mitogenic cysteinyl leukotriene 1 receptor (CysLT_1_R) and the differentiation-promoting cysteinyl leukotriene 2 receptor (CysLT_2_R) is lost. Further, our previous data indicate that patients with high CysLT_1_R and low CysLT_2_R expression have a poor prognosis. In this study, we examined whether the balance between CysLT_1_R and CysLT_2_R could be restored by treatment with the cancer chemopreventive agent all-*trans* retinoic acid (ATRA).

**Methods:**

To determine the effect of ATRA on CysLT_2_R promoter activation, mRNA level, and protein level, we performed luciferase gene reporter assays, real-time polymerase chain reactions, and Western blots in colon cancer cell lines under various conditions.

**Results:**

ATRA treatment induces CysLT_2_R mRNA and protein expression without affecting CysLT_1_R levels. Experiments using siRNA and mutant cell lines indicate that the up-regulation is retinoic acid receptor (RAR) dependent. Interestingly, ATRA also up-regulates mRNA expression of leukotriene C_4_ synthase, the enzyme responsible for the production of the ligand for CysLT_2_R. Importantly, ATRA-induced differentiation of colorectal cancer cells as shown by increased expression of MUC-2 and production of alkaline phosphatase, both of which could be reduced by a CysLT_2_R-specific inhibitor.

**Conclusions:**

This study identifies a novel mechanism of action for ATRA in colorectal cancer cell differentiation and demonstrates that retinoids can have anti-tumorigenic effects through their action on the cysteinyl leukotriene pathway.

## Background

Individuals with inflammatory bowel diseases (IBD) have a 30-50% increased risk of developing colorectal cancer [1,2]. The pro-inflammatory cysteinyl leukotrienes (CysLTs) LTC_4_, LTD_4_, and LTE_4_ are derived from arachidonic acid through the actions of 5-lipoxygenase and leukotriene C_4_ synthase (LTC_4_S) [3]. The CysLTs can induce smooth muscle constriction, vascular leakage, and eosinophil recruitment in inflammatory diseases such as asthma and rhinitis (reviewed in [4]). High levels of leukotrienes have been detected in urine from patients with IBDs including ulcerative colitis and Crohn’s disease [5,6], and treatment with the 5-lipoxygenase inhibitor Zileuton significantly alleviates IBD symptoms [7]. Importantly, an increased risk for colorectal cancer has been observed in IBD patients [2].

CysLT signaling is initiated when a ligand binds one of the two different G-protein-coupled receptors: CysLT_1_R, CysLT_2_R [8,9]. Activation of the CysLT_1_R triggers signaling through either or both the Gq- and the Gi-protein depending on the cell type, most commonly through Gq [10-12]. We have shown that LTD_4_ via CysLT_1_R can induce both Erk phosphorylation and protein kinase C activation that is involved in the regulation of the calcium signal [13,14]. These activities lead to increased proliferation, survival, and phosphatidylinositol 3-kinase- and Rac-dependent migration of colorectal cancer cells [15-17]. In contrast, CysLT_2_R promotes colorectal cancer cell differentiation by increasing the activity of the intestinal brush border enzymes alkaline phosphatase and aminopeptidase N [18]. The two receptors also have opposite functions in mast cells, where CysLT_2_R negatively regulates the mitogenic responses of CysLT_1_R [19]. The combination of high CysLT_1_R expression and low CysLT_2_R expression in colon cancer specimens is correlated with poor survival prognosis and disease outcome [18,20].

Vitamin A (retinol) and its metabolites are commonly referred to as retinoids. Retinoids play important roles in embryonic development, vision, and as cancer chemopreventive agents (see review [21,22] ). All-*trans* retinoic acid (ATRA) is a potent metabolite of vitamin A and is successfully used to treat patients with acute promyelocytic leukemia [23]. In clinical trials, retinoids have also shown promising results in head and neck, skin, ovarian, prostate, and lung cancer [23]. ATRA has also had positive results in animal models for cancer. For instance, rats on a low-fat diet supplemented with vitamin A have a reduced tumor incidence [24]. Moreover, retinoids are effective in reducing azoxymethane-induced aberrant crypt foci and colon tumors in rats [25]. ATRA treatment also reduced tumor growth 40–60% in athymic mice implanted with HT-29 colon carcinoma cells [26]. In human colon cancer cell lines, ATRA is capable of inducing growth inhibition, apoptosis, and differentiation [27].

ATRA exerts its effects through heterodimers of retinoic acid receptors (RARs) and retinoid X receptors (RXRs), which are transcription factors of the nuclear receptor family [23]. All of the known RAR isoforms (α, β, and γ) are expressed in colorectal cancer cell lines [28]. The RAR/RXR heterodimers bind constitutively to retinoic acid response elements (RAREs) in promoters of genes; these are characterized by two consensus half sites [PuG(G/T)TCA] generally arranged as direct repeats separated by 2 to 5 nucleotides [23]. Upon ligand binding, coactivators of the p160 family are recruited to replace the corepressors SMRT and NCoR, and transcription is initiated [23].

We found sequences in the CysLT_2_R promoter region that were identical to RAREs reported in the literature and hypothesized that treatment of colorectal cancer cells with ATRA would affect the expression of CysLT_2_R. Furthermore, we investigated whether ATRA-induced colon cancer cell differentiation was dependent on CysLT_2_R. LTC_4_S conjugates LTA_4_ with glutathione to form LTC_4_[3], and is induced by ATRA in rat basophilic leukemia cells and associated with subsequent cell differentiation [29]. In addition to CysLT_2_R, LTC_4_S could be induced by ATRA in colon cancer cells. It is well established that retinoids are effective inducers of differentiation in cancer cells, but few studies have addressed the pathways that mediate these effects.

## Methods

### Reagents

LTC_4_ was obtained from Cayman Chemicals Co. (Ann Arbor, MI); AP 100984 was a gift from Jilly F. Evans (Amira Pharmaceuticals); and Lipofectamine 2000, Lipofectamine LTX, and Opti-MEM were from Invitrogen (Carlsbad, CA). Hybond polyvinylidene difluoride (PVDF) membranes were from Amersham Biosciences (Little Chalfont, Bucks, UK) and Mini-PROTEAN TGX gels, Immun-blot PVDF membranes and Immun Star Western C were from Biorad (Hercules, CA). The rabbit polyclonal CysLT_1_R and CysLT_2_R antibodies were obtained from Innovagen (Lund, Sweden). The antibodies RARα C-20 (sc-551), RARβ C-19 (sc-552) and Lamin B C-20 (sc-6216), were purchased from Santa Cruz Biotechnology (Santa Cruz, CA). The secondary peroxidase-conjugated goat anti-rabbit, rabbit anti-goat and anti-mouse antibodies were purchased from Dako Cytomation (Glostrup, Denmark). The caspase-3 fluorometric substrate was obtained from Upstate (Lake Placid, NY). All other reagents were obtained from Sigma Chemicals (St Louis, MO).

### Cell culture

The colon cancer cell lines Caco-2, SW480 (ATRA-sensitive), and HCT-116 (ATRA-resistant) [30] were grown in Dulbecco’s modified Eagle medium with 100 μM non-essential amino acids, RPMI 1640, and McCoy’s 5A medium, respectively. All media were supplemented with 10% fetal bovine serum (FBS), 2 mM L-glutamine, 55 IU/mL penicillin, 55 μg/mL streptomycin, and 1.5 μg/mL fungizone (Invitrogen). The cell lines were kept at 37°C in a humidified atmosphere of 5% CO_2_ and 95% air. All experiments were performed on day 4–5 after seeding and all ATRA stimulations were performed in the dark. The cells were left in 1.5% FBS or serum-free medium overnight to synchronize the cells and were subsequently treated with 1 or 10 μM ATRA, 40 nM LTC_4_, 1 μM AP 100984, and/or 2 mM sodium butyrate for the time points indicated. Inhibitors were added 30 min before ATRA stimulation. For time courses, all cells were harvested at the same time.

### Western blot

Except for siRNA experiments, whole cell lysates were used for Western blot analysis of CysLT_1_R and CysLT_2_R. Cells were harvested in Tris lysis buffer on ice supplemented with 1% (v/v) Triton X-100 and protease inhibitors and homogenized 10 times with a Dounce homogenizer and centrifuged at 200 × g for 10 min. The supernatant was centrifuged at 1000 × g for 5 min to remove cell debris. For Western blot experiments analyzing, RARα, and RARβ, a Nuclear Extraction kit (Merck Millipore) was used according to the supplier’s instruction and CysLT_2_R membrane fractions were prepared as in [18]. The Coomassie/Bradford method (Pierce) was used to determine protein content, and equivalent protein amounts for each sample were used. Gel electrophoresis and immunoblotting was performed as described in [31] and the blots were scanned in a Molecular Imager ChemiDoc XRS+ with Image Lab software (Biorad). Stripping of the membranes was performed according to the supplier’s instructions (Re-blot Plus, Merck Millipore) and reprobed in the same way.

### qPCR analysis

Cells for RNA isolation were washed twice in PBS and immediately frozen at -80°C. The cells were scraped in the lysis buffer provided in the RNeasy Plus Mini kit (Qiagen GmbH) and homogenized 10 times with a 20-G needle. The RNA was isolated and purified according to the supplier’s instructions. In short, genomic DNA was removed and RNA was bound to RNeasy spin columns, washed, and dissolved in RNase-free water. cDNA synthesis was performed using RevertAid H Minus M-MuLV reverse transcriptase and oligo(dT)_18_ primers (Fermentas, Burlington, Canada). The mRNA expression levels of CysLT_1_R, CysLT_2_R, LTC_4_ synthase, mucin-2 (MUC-2), RARα, and the endogenous reference gene HPRT-1 were quantified using Maxima^TM^ Probe qPCR Master Mix (2x) (Fermentas). The cDNA was mixed with 0.9 μM TaqMan primers and master mix and amplified at 60°C in a Mx3005P thermocycler (Stratagene). The following Taqman primer sets (Applied Biosystems) were used: CYSLTR1, Hs00929113_m1; CYSLTR2, Hs00252658_s1; MUC2, Hs00159374_m1; LTC_4_S, Hs00168529_m1; RARA, Hs00940446_m1; RARB, Hs00977140_m1; and HPRT-1, Hs99999909_m1. The samples were analyzed and normalized against HPRT-1 using the MxPro software (Stratagene).

### siRNA experiments

Transient siRNA transfections of SW480 cells were carried out according to the manufacturer’s instructions. Briefly, 3 days after seeding and at approximately 50% confluence, cells were transfected for 4–6 h in Opti-MEM with reduced-serum without antibiotics, with a mixture of Lipofectamine 2000, and 50–100 nM RARα, RARβ, or control (non-targeting) siRNA. Human RARα (sc-29465), RARβ siRNA (sc-29466), and control siRNA-A, -B, and -C (sc-37007, sc-44230, sc-44231 respectively) were from Santa Cruz Biotechnology and ON-TARGET*plus* SMARTpool L-003437-00-0005, L-003438-00-005 and ON-TARGET*plus* Non-Targeting Pool D-001810-10-05, from Dharmacon. The cells were allowed to rest for at least 24 h in complete medium, left in 1.5% FBS or serum-free medium overnight, and stimulated on day 5 as described above.

### Alkaline phosphatase activity

Alkaline phosphatase activity was measured using disodium *p*-nitrophenyl phosphate as the substrate. Caco-2 cells were seeded in Petri dishes and incubated for 24 h at 37°C in complete Dulbecco’s modified Eagle medium that was ultraviolet-treated to remove any traces of endogenous retinoids. ATRA (1 μM) and/or AP 100984 (1 μM) were added and the cells were incubated for a total of 72 h at 37°C. Every 24 h, the medium was renewed and ATRA and/or AP 100984 were added as before. Sodium butyrate (2 mM) was used as a positive control (data not shown). Five replicates per sample of scraped and lysed cells (PBS, 0.5% Triton X-100) were added to a 96-well plate. The alkaline phosphatase activity was estimated after incubation with disodium *p*-nitrophenyl phosphate for 30 min at 37°C by measuring the absorbance at 405 nm due to formation of *p*-nitrophenol. The assay was performed as previously described in [32]. The samples were normalized for equal protein content.

### ELISA LTC_4_

SW480 cells were grown for 5 days in normal medium containing 10% serum after which the medium was changed to 1.5% serum containing medium and treated with or without 1 μM ATRA for 24 h. The media were then collected and separated by solid-phase extraction. LTC_4_ from the samples were measured using the LTC_4_ ELSA kit from Cayman.

### Thymidine incorporation assay

Five thousand SW480 cells per well were seeded and cultured for 2 days in flat-bottomed, 96-well plates. Cells were serum starved overnight and subsequently stimulated for 48 h with 1 μM ATRA in the presence or absence of 1 μM AP 100984 (CysLT_2_R inhibitor) or with medium containing 10% serum as a positive control for proliferation. Cellular DNA synthesis was assessed by adding 0.5 μCi ^3^H-thymidine (GE Healthcare) during the final 18 h of stimulation. The cells were washed once with PBS and incubated with 0.05% trypsin-EDTA solution for 10 min at 37°C. Cells were harvested, collected on filter paper, and ^3^H thymidine incorporation was measured in a 1450 Microbeta Trilux liquid scintillation counter (Wallac, Turku, Finland).

### Caspase-3 activity

SW480 cells were cultured in 6-well plates for 4 days. Cells were incubated overnight in medium containing 1.5% serum and subsequently stimulated for 48 h with 1 μM ATRA in the presence or absence of 1 μM AP 100984. Taxol (100 nM) was used as a positive control for apoptosis. The cells were lysed for 15 min on ice in 300 μL buffer containing 1% (v/v) Triton X-100, 10 mM Tris–HCl (pH 7.4), 10 mM NaH_2_PO_4_/Na_2_HPO_4_ (pH 7.5), 10 mM sodium pyrophosphate, and 130 mM NaCl. Samples (50 μL) were suspended in reaction buffer (200 μL 20 mM HEPES, 2 mM dithiothreitol, and 10% glycerol) and added to Nunc Polysorb 96-well plates where a caspase-3 fluorometric substrate, Ac-Asp-Glu-Val-Asp-AMC (3 μL; Upstate), was subsequently added. The plates were incubated at 37°C for 1 h in the dark, and the fluorescence of each well was measured at 390 nm excitation and 460 nm emission wavelengths using a BMG plate reader (Offenburg, Germany). Triplicate samples were analyzed and adjusted for equal protein content.

### Luciferase reporter assay

A *Renilla* control reporter plasmid and a pGL3-Enhancer vector with a luciferase reporter gene containing 1000 base pairs of the *CYSLTR2* gene promoter (−1 to-1012) was used for CysLT_2_R activity assays [32]. SW480 cells (5–10 × 10^4^ per well) were seeded in 12-well plates. Cells were transfected on day 3 with a mixture of the plasmids and Lipofectamine 2000 or LTX in Opti-MEM according to the supplier’s instructions. The final DNA amount per well was 1 μg for the pGL3 plasmid and 50 ng for the *Renilla* control vector. When siRNA was co-transfected, 50 nM per well was used. The transfection medium was changed to complete RPMI 1640 with 10% FBS after 5–6 h and incubated for 24 h. The medium was changed to serum-free or serum-low (1.5%) medium and cells were incubated overnight before stimulation with 10 μM ATRA. After 48 h of ATRA stimulation, the experiments were finished by rinsing the wells twice with PBS and adding passive lysis buffer from the Dual-Luciferase Reporter Assay System from Promega (Madison, WI). The plates were placed on an orbital shaker at a slow rate for 30 min and frozen (−20°C) until analysis. Firefly and *Renilla* luminescence were measured on a MiniLumat LB 9506 (Berthold Technologies) according to the protocol for the Dual-Luciferase Reporter Assay System and the ratio was calculated.

### Immunofluorescence

Cells were seeded on glass cover slips and grown for 3 days before being stimulated with 10 μM ATRA for 24 h in the absence of serum. The medium was removed and after several washes with PBS the cells were fixed with 4% paraformaldehyde for 15 min and subsequently permeabilized with 0.1% Triton X-100 for 5 min. Non-specific binding was blocked with 3% goat serum in PBS for 45 min. Cells were incubated with a primary mucin-2 (MUC-2) antibody (Santa Cruz, diluted 1:50) in 1% goat serum/PBS for 1 h followed by incubation with a secondary Alexa-488 antibody (Molecular Probes, diluted 1:500) for 1 h at room temperature. After washing in PBS, the cover slips were mounted on glass slides with fluorescent mounting medium. Confocal microscopy images were recorded using Zeiss LSM 700 (Carl Zeiss Microscopy GmbH, Jena, Germany).

### Statistics

Data was analyzed using PRISM® software (GraphPad Software Inc., La Jolla, CA). One-way ANOVA; unpaired one-sample t-test was performed when samples were compared to a control set to 100% or 1. In all other cases, an unpaired t-test was performed. Values of P < 0.05 were considered statistically significant.

## Results

### ATRA treatment increases CysLT_2_R expression in colon cancer cells

**Figure 1:**
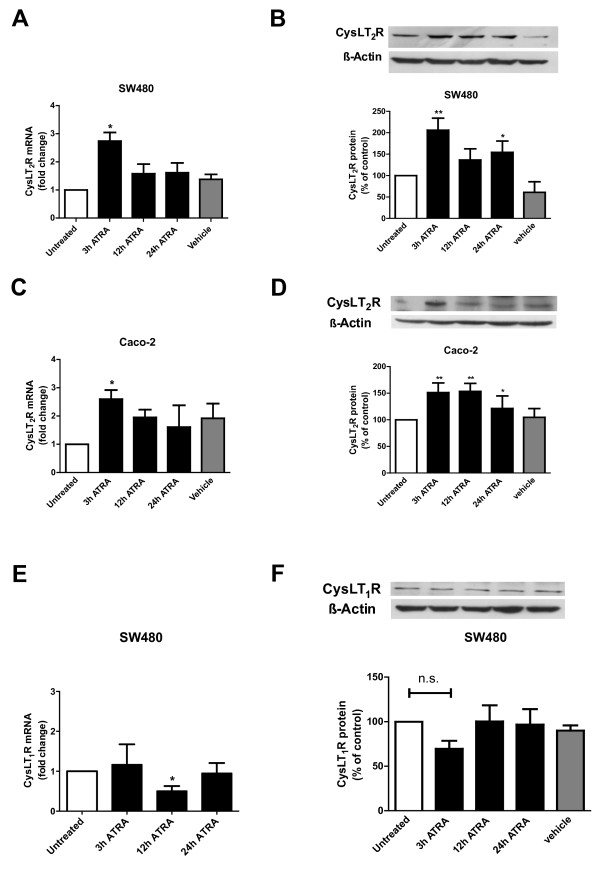
**CysLT**_**2**_**R expression in ATRA-treated colon cancer cell lines.** Cells were incubated for 3, 12, or 24 h in the absence or presence of 1 μM ATRA as indicated. **(A** and **C)** qPCR for CysLT_2_R expression. Data are normalized to HPRT-1 in SW480 and Caco-2 cells. **(B** and **D)** Protein expression of CysLT_2_R normalized to β-actin in SW480 and Caco-2 cells as determined by Western blot. **(E)** qPCR for CysLT_1_R expression in SW480 cells. Data are normalized to HPRT-1. **(F)** Protein expression of CysLT_1_R normalized to β-actin in SW480 cells as determined by Western blot. Changes in CysLT_2_R expression relative to basal levels in unstimulated cells are shown as mean ± standard error of the mean (SEM; *P < 0.05, **P < 0.01; n.s. = not significant).

ATRA is an established differentiation-inducing agent of epithelial cells [33] and we previously found that CysLT_2_R signaling also induces differentiation of colon cancer cells [18]. When either SW480 or Caco-2 colon cancer cells were stimulated with 1 μM ATRA, CysLT_2_R mRNA was induced 3 h after treatment (Figure 1A and C). Protein levels of CysLT_2_R also increased significantly, peaking at 3 h in SW480 cells and between 3–12 h in Caco-2 cells (Figure 1B and D). Because CysLT_2_R has been suggested to have opposing activities to those of CysLT_1_R, we next investigated the effect of ATRA on CysLT_1_R [18,19]. Unlike CysLT_2_R, which seems to play a role in differentiation, CysLT_1_R has mitogenic and pro-survival effects [15,16]. High CysLT_1_R expression correlates with poor prognosis of colorectal cancer patients [20]. We stimulated SW480 cells with 1 μM ATRA for 3–24 h, but failed to observe an induction in CysLT_1_R expression, at either the mRNA or protein level (Figure 1E and F).

### RARα knockdown decreases ATRA-induced CysLT_2_R mRNA and protein expression

**Figure 2:**
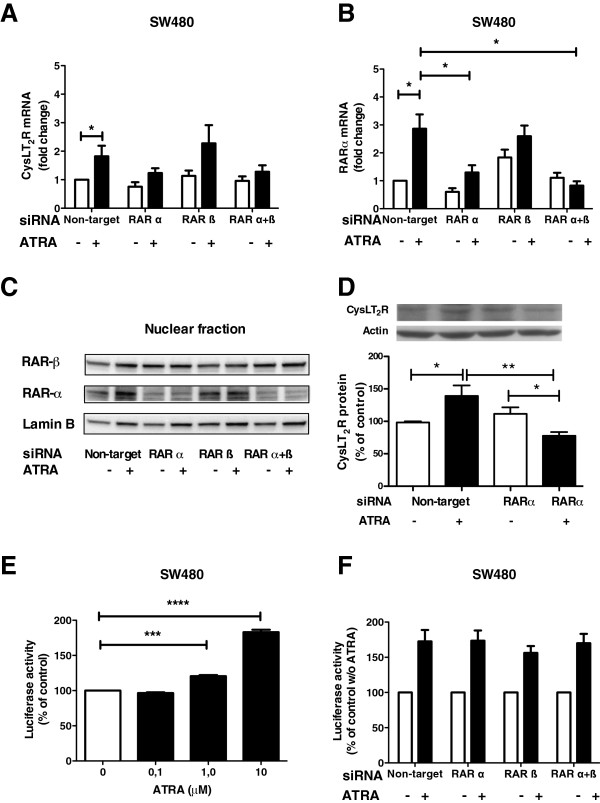
**Effect of RARα or RARβ inhibition on CysLT**_**2**_**R expression and promoter activity.** SW480 cells were transfected with control siRNA (non-target siRNA), RARα siRNA, or/and RARβ siRNA, allowed to rest for 24 h, serum-starved overnight, and subsequently stimulated with 10 μM ATRA. **(A** and **B)** qPCR for CysLT_2_R and RARα expression. Data are normalized to HPRT-1 with and without ATRA stimulation for 3 h. **(C)** Western blots without and with ATRA stimulation for 3 h. **(D)** Western blot analyzes of CysLT_2_R protein expression in membrane fractions from SW480 cells stimulated or not with ATRA for 3 h normalized to unstimulated control. **(E)** CysLT_2_R luciferase promoter activity in cells stimulated with or without ATRA for 48 h. Data are normalized to unstimulated controls. **(F)** CysLT_2_R luciferase promoter activity in siRNA-treated cells stimulated with or without ATRA for 48 h. Data are normalized to unstimulated control. Values are shown as mean ± SEM (*P < 0.05, **P < 0.01, ***P < 0.001, ****P < 0.0001).

RARs are nuclear hormone receptors for ATRA, which upon ligand binding enable the transcription of target genes [33]. Transfection of SW480 cells with siRNA against RARα, but not RARβ decreased the induction of CysLT_2_R mRNA in response to ATRA stimulation (Figure 2A). qPCR analysis showed that RARα siRNA downregulated RARα mRNA levels to approximately 50% (Figure 2B). The mRNA expression of RARβ when treated with RARβ siRNA was downregulated to the same extent (50%) but was less specific, downregulating RARα mRNA to a similar level (data not shown). We used the nuclear fraction for RARs (Figure 2C) and membrane fraction for CysLT_2_R (Figure 2D) protein detection. The induction of CysLT_2_R protein in the membrane fraction by ATRA was also abolished when siRNA against RARα was added (Figure 2D). The effect of siRNA treatment on the respective protein levels was similar to that observed for the mRNA (i.e., no or little induction after ATRA stimulation; Figure 2A-D).

To study CysLT_2_R promoter activity, SW480 cells were transiently transfected with a luciferase reporter gene vector and stimulated with ATRA. A dose response was observed with increasing ATRA concentration (0.1, 1.0, and 10 μM; Figure 2E). Stimulation with 10 μM ATRA was chosen for the inhibition studies in which cells were treated with siRNAs targeting the RARs. Neither RARα siRNA nor RARβ siRNA induced any significant change in basal CysLT_2_R promoter activity (Figure 2F). However, in contrast to the activation seen for the regulation of the endogenous CysLT_2_R gene (Figure 2D), siRNA knockdown of RARα or RARβ or a combination of the two did not affect the ATRA-induced activation of the transfected partial/putative CysLT_2_R promoter (Figure 2F).

### ATRA does not affect the proliferation of SW480 colon cancer cells

**Figure 3:**
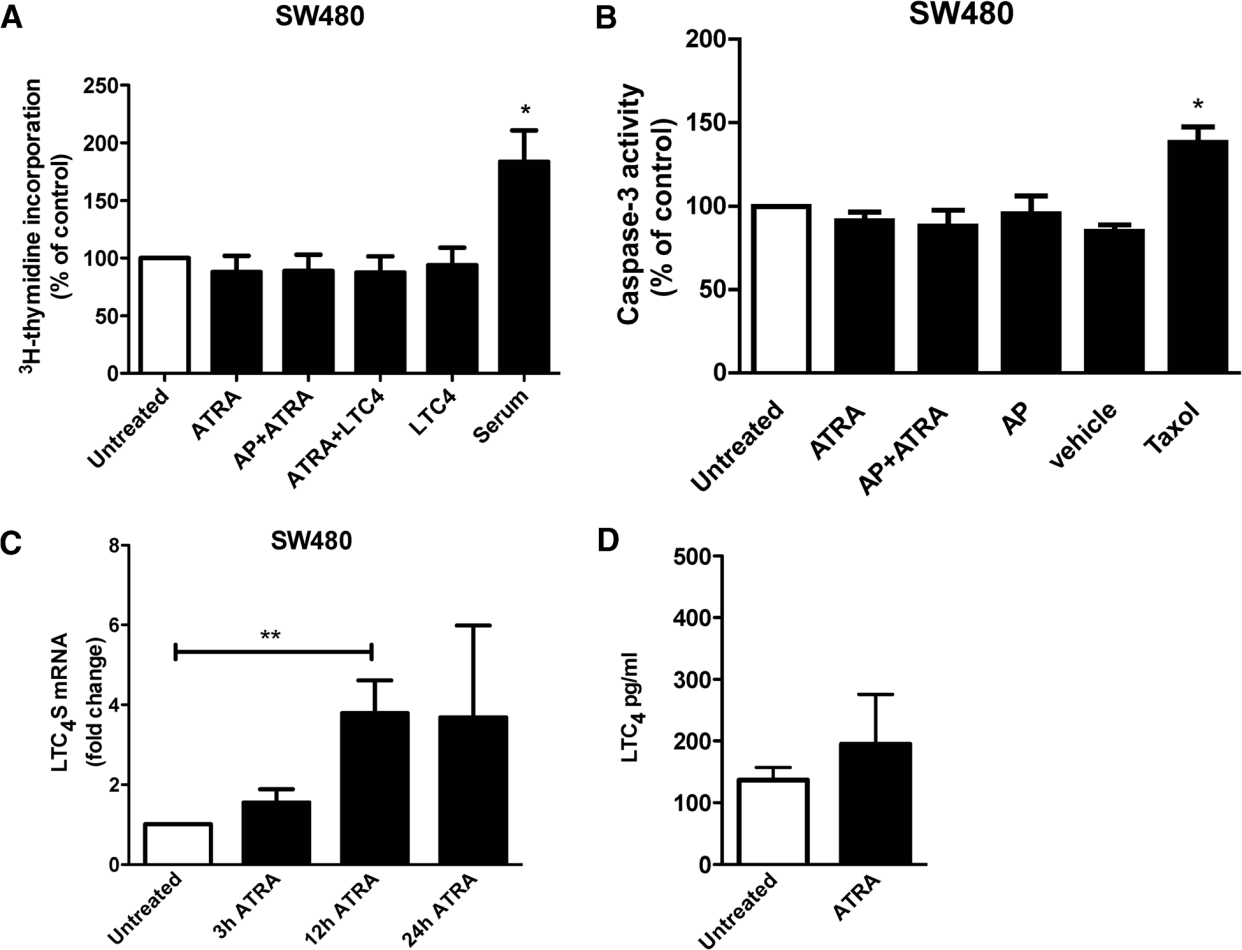
**Effect of ATRA on cell growth, apoptosis, LTC**_**4**_**S and LTC**_**4 **_**expression.** SW480 cells were treated with the CysLT_2_R inhibitor AP 100984 (AP) and/or 40 nM LTC_4_ and were or were not stimulated with 1 μM ATRA. **(A) **^3^H-thymidine incorporation measured after 72 h of ATRA treatment, normalized to unstimulated control. **(B)** Caspase-3 activity after 48 h of ATRA treatment normalized to unstimulated control. **(C)** qPCR for LTC_4_S expression after 3–24 h of ATRA stimulation. Data are normalized to HPRT-1. **(D)** Release of LTC_4_ into the media form SW480 cells after 24 h incubation in the absence or presence of ATRA. Values are shown as mean ± SEM (*P < 0.05, **P < 0.01).

The effects of ATRA on tumor suppression cannot be entirely attributed to its role in differentiation, as ATRA has also been reported to inhibit growth of some colon cancer cell lines [27,34]. To determine whether ATRA has such an activity in our system, we pre-incubated SW480 cells with or without 1 μM CysLT_2_R antagonist AP 100984 and stimulated the cells with 1 μM ATRA or 40 nM LTC_4_ for 48 h. DNA synthesis was measured as the amount of ^3^H-thymidine incorporated during the last 18 h of stimulation. Neither ATRA nor LTC_4_, alone or in combination, induced any changes in DNA synthesis compared to unstimulated cells (Figure 3A). Complete medium with 10% FBS was used as a positive control for proliferation and induced a 2-fold increase in ^3^H-thymidine incorporation. These data showed that neither ATRA nordoes the CysLT_2_R inhibitor AP 100984 have any effect on SW480 cell growth.

### Effects of ATRA on apoptosis, LTC_4_S mRNA and LTC_4_ expression in SW480 cells

In some cell types, ATRA induces apoptosis through the caspase-3 pathway [35]. We therefore investigated whether ATRA could induce apoptosis in these colon cancer cells. The cells were incubated with or without 1 μM CysLT_2_R inhibitor AP 100984 and stimulated with 1 μM ATRA for 48 h. Under these conditions, we were unable to observe apoptosis in SW480 cells as measured by caspase-3 activity (Figure 3B). Taxol was used as a positive control for apoptosis and induced a significant (50%) increase in caspase-3 activity. Neither AP 100984 alone or in combination with ATRA had any effect on caspase-3 activity, indicating that AP 100984 had no intrinsic apoptotic effect.

We next investigated whether ATRA could increase LTC_4_S mRNA expression. Cells were stimulated with ATRA for 3, 12 or 24 h and the LTC_4_S mRNA level was determined with qPCR. We observed a 4-fold increase of LTC_4_S mRNA in cells treated with ATRA for 12 h compared to control cells (Figure 3C). The induction of LTC_4_S can enhance LTC_4_ production and in turn induce CysLT_2_R activation, thus creating a positive feedback loop that promotes differentiation. Therefore, we next examined the endogenous synthesis and release of LTC_4_ in SW480 cells, we found a basal release of 140 pg/ml LTC_4_ and a possible enhanced release by ATRA to 190 pg/ml LTC_4_ in SW480 cells (Figure 3D).

### ATRA does not induce CysLT_2_R expression in ATRA-resistant HCT-116 cells

**Figure 4:**
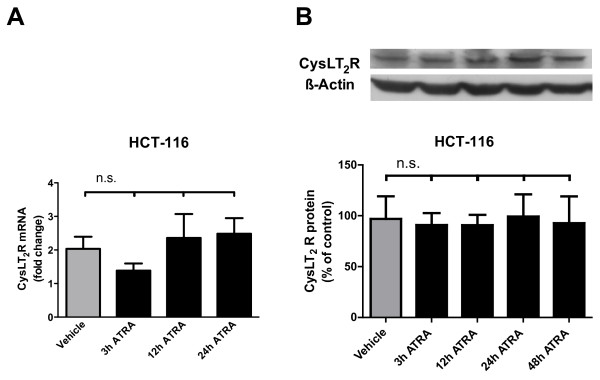
**ATRA does not affect CysLT**_**2**_**R in HCT-116 cells.** RAR-mutated HCT-116 colon cancer cells were incubated for 3, 12, or 24 h in the presence or absence of 1 μM ATRA. **(A)** qPCR for CysLT_2_R expression in HCT-116 cells. Data are normalized to HPRT-1. **(B)** Protein expression of CysLT_2_R normalized to β-actin in HCT-116 cells as determined by Western blot. Data show changes in expression relative to basal levels in unstimulated cells. (n.s. = not significant).

The colon cancer cell line HCT-116 is ATRA-resistant [34]. We confirmed this with qPCR, finding that stimulation of HCT-116 cells with 1 μM ATRA failed to induce mRNA expression of CysLT_2_R at any of the time points observed (Figure 4A). Likewise, Western blots of lysates harvested from cells treated with 1 μM ATRA for 3, 12, and 24 h showed there was no effect on CysLT_2_R protein expression (Figure 4B). In case the effect on the protein level was delayed, we also tested after 48 h of stimulation, but no significant change from the unstimulated cells was observed.

### The CysLT_2_R antagonist AP 100984 reduces ATRA-induced MUC-2 expression and alkaline phosphatase activity

**Figure 5:**
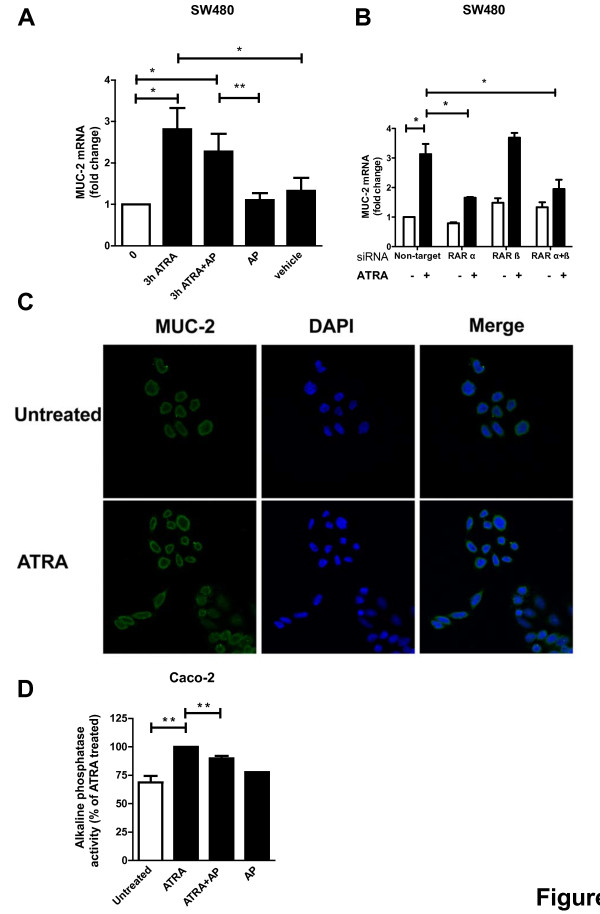
**Effect of ATRA on the differentiation markers MUC-2 and alkaline phosphatase activity.** SW480 cells were either 1) treated with 1 μM of the CysLT_2_R inhibitor AP 100984 (AP) and were or were not stimulated with 1 μM ATRA or 2) treated with RAR siRNA and stimulated with 10 μM ATRA. **(A** and **B)** qPCR for MUC-2. Data are normalized to HPRT-1 and changes in MUC-2 expression are relative to basal levels in untreated cells. **(C)** Confocal immunofluorescence microscopy showing ATRA-induced expression of Mucin-2 in SW480 colon cancer cells. After stimulation with 10 μM ATRA for 24 h the cells were fixed, permeabilized and stained with a Mucin-2 antibody. Left panels show Mucin-2 (MUC-2) staining, middle panels nuclear staining with DAPI and right panels merged, the graphs were taken with 40x objective. **(D)** Alkaline phosphatase activity in Caco-2 cells. Bars show % of ATRA-induces alkaline phosphatase activity and values are shown as mean ± SEM (*P < 0.05, **P < 0.01).

Mucins are secreted by colonocytes to form a mucus barrier to protect the intestinal epithelium [36]. MUC-2 is down-regulated in many colorectal cancers and is associated with the differentiated state of colonic epithelia [37]. We analyzed the expression of MUC-2 mRNA in SW480 cells and found that treatment with 1 μM ATRA increased MUC-2 mRNA expression 2-fold after 3 h of stimulation (Figure 5A). When cells were pretreated with 1 μM AP 100984, the ATRA-induced MUC-2 up-regulation was decreased. AP 100984 itself had no effect on MUC-2 mRNA expression. Similarly, when cells were treated with RARα siRNA, ATRA-induced MUC-2 expression was decreased by approximately 50% (Figure 5B). Thus, ATRA-enhanced MUC-2 expression in SW480 colon cancer cells is at least partly mediated through CysLT_2_R and RARα signaling. Furthermore, we also found by immunofluorescence that the MUC-2 protein expression was increased after treatment with ATRA for 24 h (Figure 5C).

Alkaline phosphatase is one of the brush border enzymes expressed in the differentiated epithelial cells of the intestines and alkaline phosphatase activity is often used as a differentiation marker [38]. In accordance with others’ results from previous studies, we found that ATRA is able to significantly induce alkaline phosphatase activity in Caco-2 cells, as determined by the formation of *para*-nitrophenol (Figure 5D) [39]. Sodium butyrate was included as a positive control for alkaline phosphatase activity, and increased the activity approximately 2-fold compared to unstimulated cells (data not shown) [40]. Interestingly, ATRA-induced alkaline phosphatase activity was significantly reduced upon pretreatment for 30 min with 1 μM CysLT_2_R inhibitor AP 100984. Addition of the inhibitor alone did not affect basal alkaline phosphatase activity, suggesting that ATRA’s induction of alkaline phosphatase activity is partly mediated through CysLT_2_R in Caco-2 cells. Together, the MUC-2 expression and alkaline phosphatase activity studies shows that ATRA’s induction of differentiation is at least partially dependent on CysLT_2_R signaling.

## Discussion

ATRA is well known to induce differentiation of epithelial cells and we have also shown that CysLT_2_R induces differentiation of epithelial cells [23,32]. We found putative RAREs within the promoter region of the transcription start of the *CYSLTR2* gene [32]. Based on this finding, we hypothesized that ATRA could induce CysLT_2_R expression. We show here that ATRA can indeed induce both mRNA and protein expression of CysLT_2_R in two different colon cancer cell lines. Knock-down of RARα with siRNA decreases ATRA-induced CysLT_2_R expression in SW480 cells. In accordance with this finding, ATRA was unable to induce CysLT_2_R mRNA or protein expression in HCT-116 colon cancer cells, which lack functional RARs. Neither mutations in these possible response elements nor truncation of the inserted region changed the cell response to ATRA stimulation (see Additional file 1: Figure S1). Furthermore, a high concentration of ATRA is required to observe any cellular response. Together, these findings might suggest that ATRA’s induction of CysLT_2_R is mediated indirectly or that the intracellular RAR levels are low. This is in agreement with findings that the relative expression of RARα is higher than RARβ in human intestine and the overall expression of RARs (especially RARβ2) is lower in tumors than normal tissue due to epigenetic modifications [28].

Previous studies showed that CysLT_2_R can be up-regulated by the cytokines interferon γ (IFNγ) and interleukin-4 in monocytes, T cells, and B cells and by interleukin-13 in monocytes [41,42]. Further, consistent with its role in inflammatory responses. Bai *et al*. [43] have shown that ATRA can down-regulate the colon inflammatory response as measured by tumor necrosis factor alpha (TNFα) levels, in patients with IBD *in vitro* and in a murine colitis model *in vivo*. We previously found that TNFα also down-regulates CysLT_2_R while up-regulating CysLT_1_R in colon cancer cells [44], an observation that also highlights the importance of maintaining receptor balance in epithelial cells [18].

ATRA has previously been shown to induce mRNA expression, protein expression, and promoter activity of LTC_4_S in rat basophilic leukemia cells. LTD_4_ is a ligand for CysLT_1_R that up-regulates both LTC_4_S and CysLT_2_R in intestinal epithelial and colon cancer cells [44]. These activities are associated with differentiation, but the underlying signaling mechanism remained unclear [29]. We show for the first time that ATRA is capable of up-regulating LTC_4_S mRNA in epithelial cells. LTC_4_S is responsible for the production of the cysteinyl leukotriene LTC_4_. Furthermore, ATRA by inducing both CysLT_2_R and possibly its ligand, activates a signaling pathway that has beneficial effects on colon epithelial cell differentiation.

MUC-2 and brush border enzymes are typical markers of differentiated colonocytes [45]. We have previously shown that LTC_4_ stimulation of CysLT_2_R induces differentiation in Caco-2 colon cancer cells, as measured by increased MUC-2 mRNA expression and increased activity of the brush border enzymes alkaline phosphatase and aminopeptidase N [18]. ATRA has also been shown to induce MUC-2 protein via PKCα and CREB in airway human tracheobronchial epithelial cells [46]. Furthermore, rats on a retinoid-deficient diet have decreased MUC-2 mRNA expression in the jejunum, ileum, and colon [47]. These studies indicate that ATRA and CysLT_2_R have similar functions in epithelial differentiation, as evidenced by MUC-2 expression. In the current study, we show that ATRA’s ability to induce MUC-2 expression in SW480 colon cancer cells might also involve CysLT_2_R signaling, as the effect can be reduced by either a CysLT_2_R inhibitor or by RARα siRNA alone or a combination of RARα and RARβ siRNA. Moreover, we found that ATRA-induced alkaline phosphatase activity could be reduced by pretreatment with the same CysLT_2_R inhibitor. Our findings indicate that CysLT_2_R signaling, likely in concert with other pathways, contributes to ATRA’s differentiation-inducing activity.

In addition to being a powerful differentiation-inducing agent, ATRA can inhibit growth in some colon cancer cell lines [48]. Therefore, we investigated whether ATRA primarily affects differentiation in SW480 cells, or also inhibits growth. In HT-29 cells ATRA-induced growth inhibition is mediated by RARα [49], whereas, in Caco-2 cells it is mediated by RARβ [50]. However, other studies have reported that ATRA has no effect on growth inhibition in HT-29 cells [27]. In certain contexts, ATRA also has the ability to mediate apoptosis [35]. In our study, we were unable to observe any effects of ATRA on growth inhibition or apoptosis in SW480 cells, suggesting that ATRA’s main activity in these cells is to induce cell differentiation.

Although the *CYSLTR2* gene has been mapped to chromosome 13q14, a region linked to atopic asthma [9], it remains unclear how the gene is regulated. When studying the promoter region of CysLT_2_R, we found a binding site for IRF-7 that showed reporter gene activity upon IFNα stimulation [32]. This finding encouraged us to further investigate whether other regulatory elements in the region were present. This analysis led us to identify, for the first time, putative RARE elements in the promoter region of CysLT_2_R. Stimulation with ATRA increased CysLT_2_R promoter activity in a reporter gene assay, but neither mutations nor truncations in the RARE elements decreased the activity. In the present study, we found a discrepancy between the regulation of the endogenous CysLT_2_R gene activity and the regulation of a transfected partial/putative promoter of the same gene. At present we do not know the reason for this discrepancy, but a couple of explanations can be considered. First, the transfected CysLT_2_R promoter is expressed at a level that in comparison with the endogenous promoter is significantly higher and therefore relatively low level of RARs do not have the same ability to regulate its activity as they have when only the endogenous promoter for CysLT2R is present. Secondly, another possibility is that our transfected putative CysLT_2_R promoter is lacking crucial bindings sites for some enhancer/cofactor that is vital for its proper regulation. Thirdly, in addition to direct ligand-dependent transcription of genes, there can be indirect effects such transactivation of other transcription factors independently of any RAR and also non-genomic mechanisms of action of ATRA [51]. Finally, our data might suggest that the effect of ATRA on CysLT_2_R promoter activity is indirect and does not involve these putative RARs. Clearly, this issue requires extensive future work before it can be resolved.

Most of the studies investigating the role of RAREs in promoters have focused on proteins involved in retinoid transport or catabolism [52,53] or developmental regulation, where ATRA is considered a ‘master switch’ for differentiation. For example, ATRA induces *hoxb1* expression, a gene responsible for gut development in mouse embryos [54]. Other ATRA-induced transcription factors or cofactors include STAT-1, Oct3/4, Hoxa1, and Hoxb4 [53,55]. Retinoids also have the ability to repress genes. In a mouse epidermal cell line, retinoids block tumor promotion by inhibiting AP-1 [56]. Interestingly, we previously observed that signaling through CysLT_1_R induces activation of the AP-1 pathway in intestinal epithelial cells, leading to increased proliferation [57]. Here we show that expression of CysLT_1_R is unaffected by ATRA treatment. High CysLT_1_R expression in cancer patients is not restricted to colorectal cancer cells; it has also been observed in transitional cell carcinoma of the bladder [58], neuroblastoma [59], astrocytoma [60], and in classical Hodgkin’s lymphoma [61]. ATRA’s mechanism of action on cysteinyl leukotriene receptors may also highlight a signaling pathway that contributes to other cancer types.

We show for the first time that in human colon cancer cells, the differentiation agent ATRA acts in part by inducing both LTC_4_S, an enzyme responsible for the generation of CysLTs, and CysLT_2_R, a receptor for these ligands. Furthermore, we report that this effect is very likely mediated through RARα or a combination of RARα and RARβ and presumably acts through a mediator that can regulate CysLT_2_R. ATRA-induced differentiation can partially be reduced by a CysLT_2_R inhibitor, implying that CysLT_2_R contributes to this differentiation. Finally, ATRA does not induce expression of the pro-mitogenic CysLT_1_R.

## Conclusions

We suggest here a mechanism by which ATRA induces differentiation, in part by increasing CysLT_2_R expression. Our data shed new light on how ATRA exerts its effects on colorectal cancer cell differentiation and demonstrates that retinoids are able to delicately regulate the balance between different elements in the cysteinyl leukotriene pathway. Further work is necessary to elucidate the interplay between retinoids and eicosanoids, but the knowledge gained from such studies could yield new insights for designing colon cancer therapy regimens.

## Competing interests

The authors declare that they have no competing interests.

## Authors’ contributions

AB, GJ and AS conceived and designed the study. AB, GJ, CA, TS and CM performed the experiments and analyzed the data. AB, GJ, and AS drafted the paper. All authors read and approved the final manuscript.

## Pre-publication history

The pre-publication history for this paper can be accessed here:

http://www.biomedcentral.com/1471-2407/13/336/prepub

## Supplementary Material

Aditional file 1: Figure S1 CysLT_2_R luciferase activity in SW480 cells transfected with a vector containing mutated putative RARE sites or truncations. Click here for file
