# Supplementary material for: The cysteinyl leukotriene 2 receptor contributes to all-trans retinoic acid-induced differentiation of colon cancer cells
Source: BMC Cancer. 2013 Jul 8;13:336. doi: 10.1186/1471-2407-13-336 (PMC3710469; doi:10.1186/1471-2407-13-336)

**Figure S1 – No effect on activity after mutation or truncation of putative RARE sites on CysLT$_2$R promoter.**

A pGL3-Enhancer vector (Promega, Madison, WI) with a luciferase reporter gene containing one thousand base pairs upstream the transcription start site (-1 to-1012) of the *CysLTR2* gene was used as a control and template for mutations of the putative RARE sites. The mutations were carried out using QuikChange XL and Multi Site-Directed Mutagenesis kits (Agilent Technologies, La Jolla, CA) with the following primers (RAREs underlined and mutations in bold).

Forward 1:

5'-CATGTTGGCCAGACTGGTCT**AA**AACTCC**AA**ACCTCAGGTGATCTGC-3',

Forward 2:

5'-GGTATTCATGTCAACAGGGT**AT**GTAGGT**AT**CAAGTTCTCTAAGTTTGAAGCGTC-3',

Reverse 1:

5'-GCAGATCACCTGAGGT**TT**GGAGTT**TT**AGACCAGTCTGGCCAACATG-3', and

Reverse 2:

5'-AAACTTAGAGAACTTG**AT**ACCTAC**AT**ACCCTGTTGACATGAATAC-3'.

We also used truncated forms of the same vector with the sequences from -1 to -187 (IRF*), from -1 to -412 (+IRF) base pairs upstream transcription start site, and from -412 to -1012 (-IRF), that contained no RARE, first or second RARE only, respectively, published earlier [1].

1. Magnusson C, Bengtsson AM, Liu M, Liu J, Ceder Y, Ehrnstrom R, Sjolander A: **Regulation of cysteinyl leukotriene receptor 2 expression--a potential anti-tumor mechanism**. *PloS one* 2011, **6**(12):e29060.

**Sequence upstream (1012 bp) from the transcription start site for the *CYSLTR2* gene with the putative retinoic acid response elements, RAREs, underlined**

GTTTCAAAACATTAAATGTAACTTAGACTCTAAAAGGGAAGGACAGCCTATTGTATTTACACA
TATTTTGGCTTACCATGTTCTTTCTTATTTTCTAATATTCTAAGTTTCCTTCTTTTATAAATTCC
TTTCTGTTTAGAGAACTTCCTTTAGTCATTCTTTGTTTGTTTGTTTTTTGTTTTGTTTTGTTGTT
GTTTTTTTTTTTTTTGAGATGGAGTTTCGCTCTTGTTGCCCCAGCTGGAGTGCAATGGCATGAT
CTCGGCTCATTGCAACTTCTGCCTCCCAGGTTCAGGCAATTCTCCTGCCTCAGCCTCCCGGGT
AGCAGAGGTGACAGGTGCCTGCCACTGGGCCCGCTAATTTTTGTATTTTTAGTAGAGACGGAG
TTTCACCATGTTGGCCAGACTGGTCT**TGAACT**CC**TGACCT**CAGGTGATCTGCCAGCCTCAGC
TTCCCAAACTGCTGGGATTACGGGCGTGAGCCACCAAACCCAGCCTAGTCATTCTTTTAAGAT
AGGTCTGCTGGCAGCAAAGTCTTAGTTTTCCGTCTGAAAAAGTCTTGATTTTTCTCTTTGTACT
TGAAGGATATTTTTGCTGGACATAGAATACTAGTTTGGCAGGTCTTTTCTTTCAGCATTTGAG
AAATGTGCCATTTCTTTCTGTCCTCCATAGTTTCTGATGAGAAATCTGCTATTCAGTAGTTTTA
GGCTGCAATATCATAGTAAGGTATTCATGTCAACA**GGGTCA**GT**AGGTCA**CAAGTTCTCTAAG
TTTGAAGCGTCAGCTTCAACCAAACAAATTAATGGCTATTCTACATTCAAAAATCAGGAAATT
TAAATTTATTATGAAATGTAATGCAGCATGTAGTAAAGACTTAACCAGTGTTTTAAAACTCAA
CTTTCAAAGAAAAGATAGTATTGCTCCCTGTTTCATTAAAACCTAGAGAGATGTAATCAGTAA
GCAAGAAGGAAAAAGGGAAATTCACAAAGTAACTTTTTGTGTCTGTTTCTTTTTAACCCAGC

**Truncations of $CysLT_2R$ promoter**

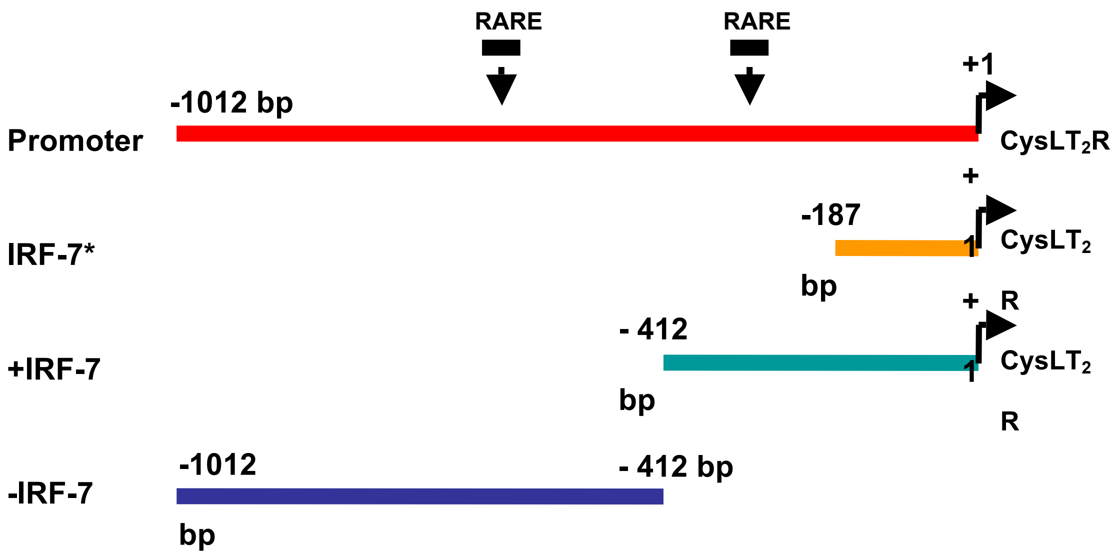


(**A** and **B**) Luciferase activity of cells transfected with plasmids with mutated RARE sites (first, second or both, respectively), or with truncated inserts. Stimulation with 10 µM ATRA for 48 h shows no change in the increase in promoter activity compared to the unstimulated plasmid. These results indicate that the effect of ATRA is indirect.

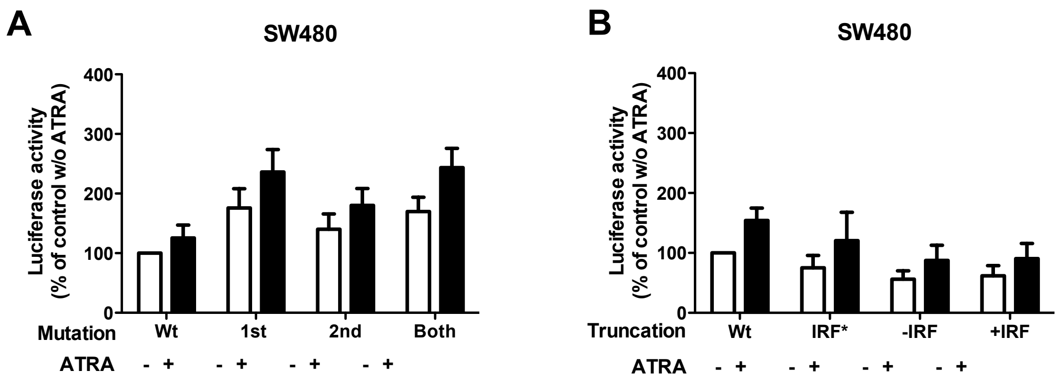

Supplement: Aditional file 1: Figure S1 — CysLT2R luciferase activity in SW480 cells transfected with a vector containing mutated putative RARE sites or truncations. [file 1471-2407-13-336-S1.pdf]
